# Increasing preoperative body size in breast cancer patients between 2002 and 2016: implications for prognosis

**DOI:** 10.1007/s10552-018-1042-z

**Published:** 2018-05-26

**Authors:** Agatha Wisse, Helga Tryggvadottir, Maria Simonsson, Karolin Isaksson, Carsten Rose, Christian Ingvar, Helena Jernström

**Affiliations:** 1 Division of Oncology and Pathology, Department of Clinical Sciences, Lund, Lund University, Barngatan 4, 22185 Lund, Sweden; 2 CREATE Health and Department of Immunotechnology, Lund University, Medicon Village (Building 406), 22381 Lund, Sweden; 3 Division of Surgery, Department of Clinical Sciences, Lund, Lund University and Skåne University Hospital, 22185 Lund, Sweden

**Keywords:** Breast cancer, Body mass index, Waist circumference, Breast volume, Changes over time, Prognosis

## Abstract

Overweight and obesity are increasing worldwide, but the extent in breast cancer patients is unknown. The two aims were to study secular trends in preoperative body mass index (BMI), waist circumference, and breast volume and their impacts on clinical outcome. BMI, waist circumference, and breast volume were measured preoperatively in 24–99-year-old primary breast cancer patients (*n* = 640) in Sweden 2002**–**2016. The measurements were analyzed alone and combined in relation to recurrence and overall survival (OS). BMI, waist circumference, and breast volume increased 2002–2016 (*p*_trends_ < 0.0001). Of these, a breast volume ≥ 850 mL was associated with the strongest recurrence-risk (adjusted hazard ratio [_adj_HR] 1.67; 95% CI 1.17–2.39), especially combined with waist circumference ≥ 80 cm (_adj_HR 2.07; 95% CI 1.25–3.44), while BMI ≥ 25 kg/m^2^ or large waist circumference conferred almost a twofold risk for death (both Log-Rank *p* ≤ 0.0001). Chemotherapy seemed to counteract the negative impact of a high BMI or large waist circumference on OS. Large breast volume was the strongest predictor for recurrence in all treatment groups. In conclusion, preoperative BMI, waist circumference, and breast volume increased between 2002 and 2016. Larger body size negatively impacted breast cancer-free interval and OS. If confirmed, body measurements may help select patients requiring more individualized treatment.

**Electronic supplementary material:**

The online version of this article (10.1007/s10552-018-1042-z) contains supplementary material, which is available to authorized users.

## Introduction

Approximately 39% of all adults in the world suffered from overweight or obesity in 2016 [1]. This number is increasing worldwide [2], making overweight and obesity a major public health problem. Breast cancer is the most common type of cancer in women [3]. A higher body mass index (BMI) in premenopausal women may protect against premenopausal breast cancer [4, 5] but not for triple-negative breast cancer [6]. Weight gain since age 18 and postmenopausal obesity increase the risk for postmenopausal breast cancer [4, 7, 8]. It remains to be determined whether the observed increased body size observed in the general population is also present in breast cancer patients. Several studies have reported that obesity is associated with a poor prognosis in breast cancer [9–13]. One of these studies also reported overweight being linked to increased breast cancer mortality [13]. Obesity causes local changes in the breast, such as the unregulated growth of adipocytes, which may promote cancer progression [14]. A review of observational studies found that obesity elevated breast cancer mortality especially in postmenopausal patients with estrogen receptor positive (ER+) tumors [15]. A dose–response effect between BMI and breast cancer-specific mortality has also been reported [9]. Moreover, higher BMIs may adversely impact treatment response [16, 17]. However, body composition measures such as waist-to-hip ratio (WHR) and waist circumference provide more information about fat distribution than BMI and thus may provide more prognostic information than BMI alone [18].

Measures of body composition include height, weight, BMI, breast volume, waist and hip circumferences, and WHR. Central adiposity affects breast cancer prognosis, and specifically, waist circumference is associated with all-cause mortality [19]. In a subset of the current study cohort, breast cancer patients with ER+ tumors and large breast volumes were found to have significantly shorter disease-free survival compared with patients with smaller breast volumes. Further, after taking breast volume into account, BMI and WHR were no longer prognostic. More aggressive tumor characteristics were also identified in patients with larger breasts compared to those with smaller breasts [20]. Another study found that after adjustment for BMI and other covariates, bra cup size was the strongest predictor for breast cancer mortality [21].

If the increasing rates of overweight and obesity in the general population are reflected in the breast cancer population, this could have a profound impact on the current trend of decreased mortality in breast cancer patients in high-income countries [22].

The first aim of this study was to identify trends over time in preoperative anthropometric measurements in primary breast cancer patients between 2002 and 2016. A secondary aim was to investigate whether any of these measurements differentially impacted the prognosis relative to age at inclusion, tumor ER status, or treatment group.

## Materials and methods

### Study population

**Fig. 1:**
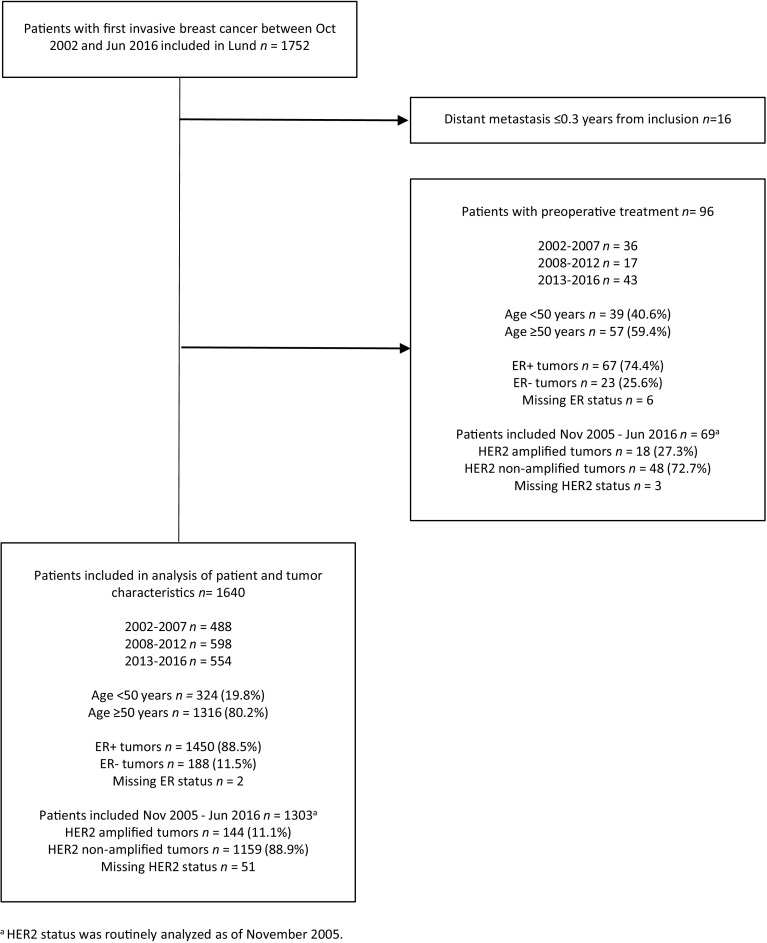
Flowchart of included and excluded patients and their characteristics

Patients with primary breast cancer at the Skåne University Hospital in Lund, Sweden, were preoperatively invited to participate in an ongoing prospective breast cancer cohort—the BC-Blood Study. Patients diagnosed with another type of cancer in the past 10 years were excluded. A total of 1,752 patients with a first invasive breast cancer were enrolled between October 2002 and June 2016. Figure 1 includes a flowchart showing the included and excluded patients. Eighteen of the originally included patients developed metastatic spread within 0.3 years of inclusion and were therefore excluded. A further 96 patients who had received preoperative treatment with either laser-thermal therapy, neoadjuvant chemotherapy, or neoadjuvant endocrine therapy were excluded because the treatments may have affected the exposures of interest. The exclusions left a total of 1,640 patients for the analysis. Written informed consent was obtained from all participants. The study was approved by the Lund University Ethics Committee (LU Dnr75-02, Dnr37-08, Dnr658-09, Dnr58-12, Dnr379-12, Dnr227-13, Dnr277-15, and Dnr458-15).

The patients preoperatively completed a questionnaire that contained questions about medication use during the past week and lifestyle factors such as smoking and alcohol consumption. The tumor characteristics for each individual patient were collected from pathology reports, which included the tumor size, axillary lymph node involvement, histological grade, and ER and progesterone receptor (PgR) status. Data concerning breast cancer events, defined as local or regional recurrence, new breast cancer, and distant metastasis, were collected from the Regional Tumour Registry, patient charts, and pathology reports. Postoperative adjuvant treatment information was obtained from patient charts and questionnaires. Treatment data were considered until the last follow-up prior to any breast cancer event or death. The date of death due to any cause was obtained from the Swedish Population Registry.

Being < 50 years of age at inclusion was used as a proxy marker for premenopausal status and compared to ≥ 50 years. Restriction analyses with a cut-off of ≥ 55 years were also performed to better reflect postmenopausal status. Body measurements were made by a trained nurse. Weight was self-reported for a small minority of patients (*n* = 41). BMI was calculated as the weight in kilograms divided by the square of the height in meters (kg/m^2^). In accordance with the WHO classification system, the cut-off for BMI was set at ≥ 25 kg/m^2^ [23]. A WHR of > 0.85 was used as an indicator for central obesity, as was waist circumference ≥ 80 cm [24]. Breast volume was measured with plastic cups as used by surgeons when performing breast surgery. A breast volume of ≥ 850 mL was selected as a cut-off value, as previously described for this cohort [20].

Patients that defined themselves as smokers, occasional smokers, or smoked > 0 cigarettes were grouped as ‘smokers.’ Based on the categories used in the Alcohol Use Disorders Identification Test (AUDIT) [25], alcohol consumption frequency was classified as follows: never, ≤ 1 time per month, 2–4 times per month, 2–3 times per week, ≥ 4 times per week.

Tumor characteristics were obtained from pathology reports. Tumors were considered ER+ when over 10% of the cell nuclei were stained [26], and this cut-off is still used in clinical practice in Sweden in 2018. Bilateral tumors were registered for 35 (2.1%) of the patients. Two patients had discordant ER status of the tumors, and for seven patients the contralateral tumor was in situ and had no ER status reported. When the tumor ER status in the left and right breast was discordant, the patient was classified as having at least one ER+ tumor.

### Statistical analysis

The statistical analyses were performed with SPSS version IBM 22.0 or 24.0 (IBM Corp., Armonk, NY, USA). To obtain the most equal distribution of the number of patients when analyzing for changes over time, the patients were distributed in the time periods 2002–2007, 2008–2012, and 2013–2016. Descriptive patient characteristics are presented as either continuous [mean and standard deviation (SD)] or categorical (number or percentage) variables. The BMI, WHR, waist circumference, and breast volume were not normally distributed and were transformed using the natural logarithm (Ln) to obtain a more normal distribution (geometric means and geometric SD). The variables were also dichotomized as follows: age at inclusion (≥ 50 years), BMI (≥ 25 kg/m^2^), WHR (> 0.85), waist circumference (≥ 80 cm), breast volume (≥ 850 mL), current smoker prior to inclusion (yes), alcohol abstention prior to inclusion (yes), adjuvant treatment with chemotherapy (yes), radiotherapy (yes), aromatase inhibitors (AIs; yes), or tamoxifen (yes). Since HER2 amplification was not routinely analyzed prior to November 2005, trastuzumab treatment was coded as (no, yes, included prior to November 2005). Tumor characteristics were analyzed as invasive tumor size (1–20 vs. ≥ 21 mm or any skin or muscular involvement), any axillary lymph node involvement (yes), histological grade III (yes), ER, PgR, and HER2 (amplified/non-amplified) status. Generalized linear models were used to obtain age-adjusted geometric means or mean percentages with 95% Wald confidence intervals (CI) via estimated marginal means for the above variables for each time period. Potential changes in the patient and tumor characteristics over time were measured using age-adjusted linear regression (continuous variables) or age-adjusted logistic regression (dichotomous variables), for which age-adjusted *p*_trend_ values are presented, except for age where crude *p*_trend_ values are presented. Effect modifications by ER status and age were tested through formal interaction analyses, where BMI ≥ 25 kg/m^2^, waist circumference ≥ 80 cm, and breast volume ≥ 850 mL were multiplied by ER status or age, respectively.

For the breast cancer-free interval analyses, patients were followed from inclusion in the study until a first breast cancer event, the last follow-up, or death prior to July 1, 2016 occurred. For overall survival, patients were followed from time at inclusion until the last follow-up, or death prior to July 1, 2016. The Kaplan–Meier method and the Log-Rank test were used to determine the breast cancer-free interval and overall survival.

Crude and adjusted Cox regression analyses were used to obtain hazard ratios (HRs), for which are presented as HRs with their 95% confidence intervals (CIs). In the case of bilateral tumors, the most aggressive tumor was selected based on the axillary lymph node, invasive size, and histological grade. HRs were determined for four different adjustment models: Model 1: age at inclusion (continuous), and tumor characteristics (invasive tumor size < 21 vs. ≥21 mm or skin or muscular involvement independent of size), any axillary lymph node involvement, histological grade III, and ER status; Model 2: Model 1, plus alcohol abstention, and current smoking prior to inclusion; plus treatments (chemotherapy, radiotherapy, tamoxifen, AIs, trastuzumab). Model 3: Model 2 plus mutually adjusted for BMI, waist circumference, and breast volume. Power calculations including 1,500 patients, of which 50% had a BMI ≥ 25 kg/m^2^, or 80% with a waist circumference of ≥ 80 cm, with an accrual interval of 14 years and additional follow-up time of 0.5 years, 0.8 power, and *α* of 0.05 showed that with a mean survival time of 4.91 years for normal weight patients, and 5.97 years for patients with small waist circumference it was possible to detect true HRs of ≤ 0.83 or ≥ 1.21 and of ≤ 0.79 or ≥ 1.29, respectively. Power calculations including 1,300 patients, of which 60% had a breast volume ≥ 850 mL, with a mean survival time of 5.06 years for patients with smaller breasts it was possible to detect true HRs of ≤ 0.82 or ≥ 1.24. The power calculations were performed with the PS Power and Sample Size Calculation Program, version 3.1.2 [27]. Due to the exploratory nature of the study, nominal *p* values are shown without adjustments for multiple testing. All statistical tests were two-sided. A *p* value of < 0.05 was considered significant.

## Results

### Patient and tumor characteristics between 2002 and 2016

Table 1 shows the patient characteristics of the 1,640 patients included in this study, overall and stratified according to year of inclusion; 2002–2007, 2008–2012, and 2013–2016. The age at inclusion ranged from 24 to 99 years. The age at inclusion, weight, BMI, WHR, waist circumference, and breast volume increased significantly between 2002 and 2016, while the percentage of patients that currently smoked prior to inclusion decreased significantly. Height and the percentage of alcohol abstainers remained stable.

**Table 1:** Patient and tumor characteristics at inclusion and treatments

			Year of inclusion			
	All patients	Missing	2002–2007	2008–2012	2013–2016	*p*_trend_
	*n* = 1,640		488 (29.8%)	598 (36.5%)	554 (33.8%)	
	Mean (SD) or number (%)		Mean (SD) or number (%)	Mean (SD) or number (%)	Mean (SD) or number (%)	
Age at inclusion (years)	60.9 (11.4)	0	59.4 (11.0)	61.1 (11.0)	62.1 (12.0)	0.0001
Age ≥ 50 (years)	1,316 (80.2)	0	386 (79.1)	487 (81.4)	443 (80.0)	0.75

	Geometric mean (SD) or number (%)	Missing	2002–2007	2008–2012	2013–2016	Age-adjusted *p*_trend_
			Age-adjusted Geometric mean (95% Wald CI)	Age-adjusted Geometric mean (95% Wald CI)	Age-adjusted Geometric mean (95% Wald CI)	
Weight (kg)	70.4 (1.2)	89	68.5 (67.4–69.6)	71.1 (70.0-72.2)	71.5 (70.3–72.6)	0.0003
Height (cm)	165.6 (1.0)	66	165.1 (164.6–165.7)	165.9 (165.4–166.4)	165.7 (165.2–166.2)	0.17
BMI kg/m^2^	25.6 (1.2)	92	25.1 (24.7–25.5)	25.8 (25.5–26.2)	26.0 (25.6–26.4)	0.001
BMI ≥ 25 kg/m^2^ (%)	809 (52.3)	92	47.3% (42.9–51.8)	54.4% (50.2–58.5)	54.8% (50.3–59.1)	0.021
Waist-to-hip ratio	0.87 (1.1)	142	0.83 (0.83–0.84)	0.88 (0.87–0.88)	0.89 (0.89–0.90)	< 0.0001
Waist-to-hip ratio > 0.85 (%)	939 (62.7)	142	39.4% (35.1–43.8)	68.1% (64.1–71.9)	81.1% (77.2–84.4)	< 0.0001
Waist circumference	89.1 (1.2)	142	85.4 (84.4–86.4)	90.6 (89.6–91.6)	91.5 (90.3–92.6)	< 0.0001
Waist circumference ≥ 80 cm (%)	1179 (78.7)	142	68.1% (63.8–72.2)	83.3% (80.0–86.2)	85.7% (82.2–88.6)	< 0.0001
Breast volume^b^ (mL)	989 (1.9)	316	935 (881–992)	976 (924–1031)	1,062 (1000–1128)	0.003
Breast volume ≥ 850 mL (%)	800 (60.4)	316	60.1% (55.3–64.8)	55.9% (51.5–60.4)	67.0% (62.3–71.5)	0.049
Current smoker prior to inclusion (%)	289 (17.7)	8	20.7% (17.3–24.6)	18.9% (15.9–22.2)	13.2% (10.6–16.3)	0.001
Alcohol abstainer (%)	192 (11.8)	7	11.5% (10.5–16.3)	9.6% (7.5–12.2)	13.1% (10.5–16.2)	0.38
Invasive tumor size						
≥21 mm or skin or muscular involvement	423 (25.8)	0	26.7% (22.9–30.8)	28.5% (25.0–32.3)	21.9% (18.6–25.5)	0.066
Any axillary lymph node involvement, yes	562 (34.3)	2	37.8% (33.6–42.3)	38.3% (34.5–42.3)	26.7% (23.2–30.6)	0.0001
Histological grade III	454 (27.8)	6	19.0% (15.8–22.7)	30.7% (27.1–34.5)	32.2% (28.4–36.2)	< 0.0001
Hormone receptor status						
ER+	1,450 (88.5)	2	87.4% (84.2–90.1)	89.1% (86.4–91.4)	88.9% (86.0–91.3)	0.47
PgR+	1,165 (71.1)	2	69.4% (65.1–73.3)	72.6% (68.9–76.1)	71.1% (67.2–74.8)	0.56
HER2 amplification^c^	144 (11.1)	51	13.4% (9.1–19.3)	12.0% (9.6–14.9)	8.6% (6.6–11.3)	0.034
Treatments by last follow-up prior to any event						
Ever chemotherapy	453 (27.6)	1	12.0% (9.5–15.1)	32.3% (28.4–36.4)	28.7% (24.8–33.0)	< 0.0001
Ever radiotherapy	1,012 (61.7)	1	60.5% (56.0–64.8)	66.2% (62.3–69.9)	59.0% (54.8–63.1)	0.56
Ever trastuzumab^c^	110 (8.1)	0	4.9% (2.7–8.7)	10.1% (7.9–12.8)	5.5% (3.9–7.7)	0.40
ER+ only						
Ever tamoxifen	730 (50.4)	3	62.0% (57.2–66.6)	60.5% (56.2–64.7)	29.5% (25.6–33.8)	< 0.0001
Ever aromatase inhibitors	580 (40.1)	2	41.7% (37.0–46.6)	39.8% (35.6–44.1)	37.4% (33.1–41.9)	0.20

^a^Interquartile range

^b^Breast volume was not included for patients with previous breast surgery

^c^Her2 was routinely analyzed as of November 2005

With respect to tumor characteristics, patients presented with less aggressive tumors over time as indicated by a significant decrease in the frequency of node-positive patients. However, the proportion of patients with histological grade III tumors significantly increased. There was also a significant decrease in the proportion of patients with HER2-amplified tumors. Tumor size and ER and PgR status remained stable.

Regarding treatment, the proportion of patients that received chemotherapy significantly increased, while the proportion of patients treated with tamoxifen significantly decreased. The proportion of patients treated with radiotherapy, AIs, and trastuzumab remained relatively stable.

### Breast cancer-free interval in relation to BMI, waist circumference, and breast volume

Patients were followed for up to 13 years. The median follow-up time for the 1,413 patients still at risk was 3.05 years (interquartile range 1.11–7.05). During this time a total of 166 breast cancer events were registered, and 139 patients died due to any cause, 78 of whom had a prior breast cancer event registered.

**Fig. 2:**
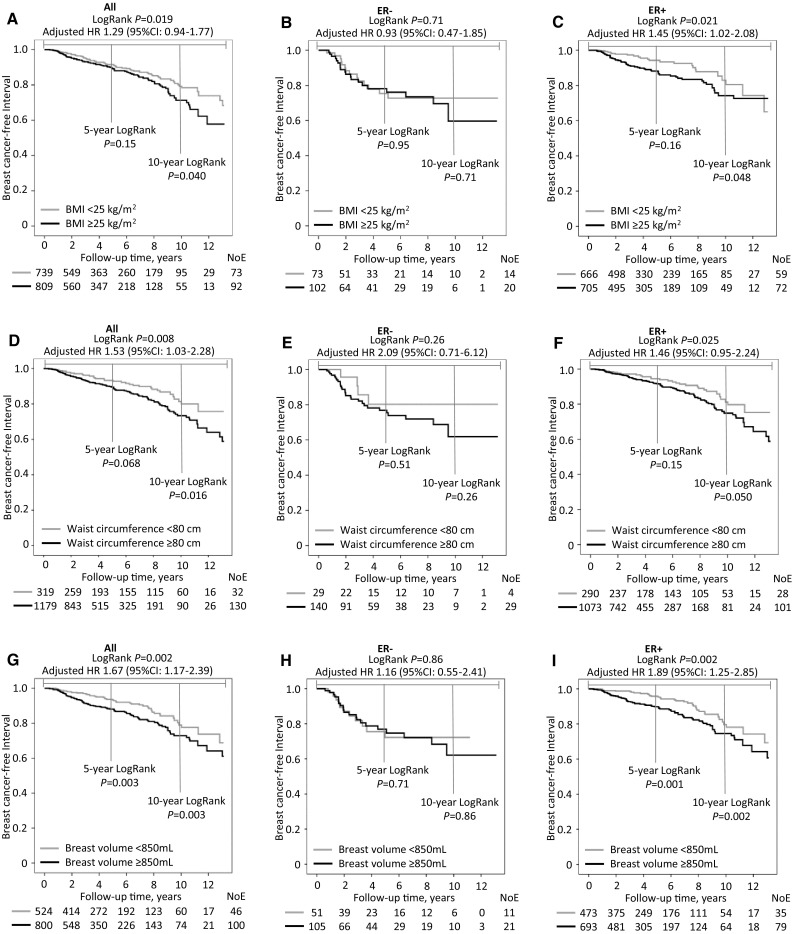
**a–i** BMI, waist circumference, and breast volume alone and stratified by ER status in relation to the breast cancer-free interval. Because this is an ongoing cohort study, the number of patients decreased with each follow-up. HRs are presented with the 95% CIs and were adjusted for age at inclusion (continuous), invasive tumor size (21 mm vs. ≥21 mm or skin or muscular involvement independent of size), any axillary lymph node involvement, histological grade III, and ER status (Model 1, Table 2)

Patients with larger body sizes as measured by BMI (≥ 25 kg/m^2^), waist circumference (≥ 80 cm), and breast volume (≥ 850 mL) had shorter breast cancer-free intervals compared to patients with lower BMIs, smaller waist circumferences and smaller breast volumes (Fig. 2a, d, g). The differences in the breast cancer-free intervals based on body-size measurements increased with follow-up time. The association between larger body sizes and shorter breast cancer-free intervals was mainly driven by the subgroup of patients with ER+ tumors (Fig. 2c, f, i). No association was found in patients with ER− tumors (Fig. 2b, e, h), although there were no significant effect modifications by ER status (all adjusted *p*_interactions_ ≥ 0.26). When stratified by age ≥ 50 years, a BMI ≥ 25 kg/m^2^ in older patients was associated with a shorter breast cancer-free interval (Log-Rank *p* = 0.021; _adj_HR 1.38; 95% CI 0.96–1.99). This was not found in the younger patients (Log-Rank *p* = 0.33); however, no significant effect modifications by age were found (all adjusted *p*_interactions_≥0.45). A waist circumference ≥ 80 cm in the older patients was associated with a shorter breast cancer-free interval (Log-Rank *p* = 0.001; _adj_HR 2.00; 95% CI 1.20–3.34), but not in the younger patients (Log-Rank *p* = 0.96). A breast volume ≥ 850 mL was associated with a shorter breast cancer-free interval in both the older (Log-Rank *p* = 0.013; _adj_HR 1.62; 95% CI 1.06–2.47) and younger (Log-Rank *p* = 0.026; _adj_HR 1.75; 95% CI 0.89–3.46) patients.

**Table 2:** Breast cancer-free interval by BMI, waist circumference, and breast volume and combinations of BMI, waist circumference, and breast volume

	Total	Events	Missing	Crude HR	Model 1	Model 2	Total	Events	Model 3
	*n* 1,640	*n*	*n*	HR (95% CI)	_adj_HR (95% CI)^a^	_adj_HR (95% CI)^abc^	*n* 1,295	*n*	_adj_HR (95% CI)^abcd^
BMI < 25 kg/m^2^	739	73	92	Ref	Ref	Ref	621	65	Ref
BMI ≥ 25 kg/m^2^	809	92		**1.44 (1.06–1.97)**	1.29 (0.94–1.77)	1.36 (0.99–1.89)	674	79	0.94 (0.62–1.41)
Waist circumference < 80 cm	319	32	142	Ref	Ref	Ref	278	29	Ref
Waist circumference ≥ 80 cm	1179	130		**1.68 (1.14–2.48)**	**1.53 (1.03–2.28)**	**1.70 (1.14–2.54)**	1017	115	1.45 (0.89–2.38)
Breast volume < 850 mL	524	46	316	Ref	Ref	Ref	515	45	Ref
Breast volume ≥ 850 mL	800	100		**1.71 (1.21–2.42)**	**1.67 (1.17–2.39)**	**1.81 (1.25–2.62)**	780	99	**1.63 (1.07–2.48)**
Combinations of BMI and waist circumference			148						
BMI < 25 kg/m^2^ and waist circumference < 80 cm	305	31		Ref	Ref	Ref	266	28	Ref
BMI ≥ 25 kg/m^2^ and waist circumference < 80 cm	13	1		0.81 (0.11–5.92)	0.52 (0.07–3.89)	0.52 (0.07–3.91)	12	1	0.38 (0.05–2.95)
BMI < 25 kg/m^2^ & waist circumference ≥ 80 cm	403	41		1.49 (0.93–2.38)	1.38 (0.86–2.22)	1.53 (0.95–2.47)	355	37	1.34 (0.80–2.25)
BMI ≥ 25 kg/m^2^ and waist circumference ≥ 80 cm	771	89		**1.78 (1.18–2.69)**	**1.55 (1.02–2.36)**	**1.72 (1.12–2.65)**	662	78	1.31 (0.78–2.20)
Combinations of breast volume and BMI			322						
Breast volume < 850 mL & BMI < 25 kg/m^2^	410	37		Ref	Ref	Ref	406	36	Ref
Breast volume ≥ 850 mL and BMI < 25 kg/m^2^	217	29		1.53 (0.94–2.49)	1.51 (0.92–2.47)	**1.75 (1.05–2.91)**	215	29	1.54 (0.90–2.62)
Breast volume < 850 mL and BMI ≥ 25 kg/m^2^	111	9		1.12 (0.54–2.32)	0.92 (0.44–1.93)	1.03 (0.49–2.19)	109	9	0.83 (0.37–1.83)
Breast volume ≥ 850 mL and BMI ≥ 25 kg/m^2^	580	71		**1.86 (1.25–2.78)**	**1.71 (1.13–2.58)**	**1.86 (1.22–2.84)**	565	70	1.49 (0.91–2.45)
Combinations of breast volume and waist circumference			317						
Breast volume < 850 mL and waist circumference < 80 cm	229	19		Ref	Ref	Ref	226	19	Ref
Breast volume ≥ 850 mL and waist circumference < 80 cm	52	10		2.10 (0.98–4.53)	1.71 (0.79–3.70)	1.91 (0.87–4.21)	52	10	1.92 (0.87–4.24)
Breast volume < 850 mL and waist circumference ≥ 80 cm	295	27		1.70 (0.94–3.06)	1.43 (0.78–2.60)	1.57 (0.86–2.88)	289	26	1.59 (0.86–2.96)
Breast volume ≥ 850 mL and waist circumference ≥ 80 cm	747	90		**2.27 (1.38–3.73)**	**2.07 (1.25–3.44)**	**2.36 (1.41–3.96)**	728	89	**2.46 (1.36–4.34)**

Events (E) and additional missing data (MD), respectively, in the adjusted models; in Model 1: BMI 164 E, 10 MD, waist circumference 161 E, 10 MD, breast volume 145 E, 9 MD, in Model 2: BMI 163 E, 25 MD, waist circumference 160 E, 24 MD, breast volume 144 E, 22 MD

Bold numbers indicate significance with a *p*-value < 0.05

^a^Adjusted for age at inclusion (continuous), invasive tumor size (< 21 mm vs. ≥ 21 or skin or muscular involvement independent of size), any axillary lymph node involvement (yes), histological grade III (yes), and ER status

^b^Adjusted for alcohol abstention (yes) and current smoking prior to inclusion (yes)

^c^Adjusted for treatment; chemotherapy, radiotherapy, tamoxifen, AIs, trastuzumab

^d^Mutually adjusted for BMI ≥ 25 kg/m^2^, waist circumference ≥ 80 cm, and breast volume ≥ 850 mL

Increased BMI, waist circumference, and breast volume were associated with shorter breast cancer-free survival in the crude model (Table 2). Waist circumference and breast volume, but not BMI, remained associated with breast cancer-free survival after adjustment for age at inclusion and tumor characteristics (Model 1), and became stronger after further adjustment for current smoking prior to inclusion, alcohol abstention, and treatments (Model 2). In general, patients with larger combined anthropometrics had the highest recurrence-risk compared to the other combined groups. Overall, breast volume was the strongest predictor for the breast cancer-free interval, both alone and combined with either BMI or waist circumference. Breast volume was the only anthropometric factor that remained significant after mutual adjustment for BMI and waist circumference (Model 3).

### Overall survival in relation to BMI, waist circumference, and breast volume

**Fig. 3:**
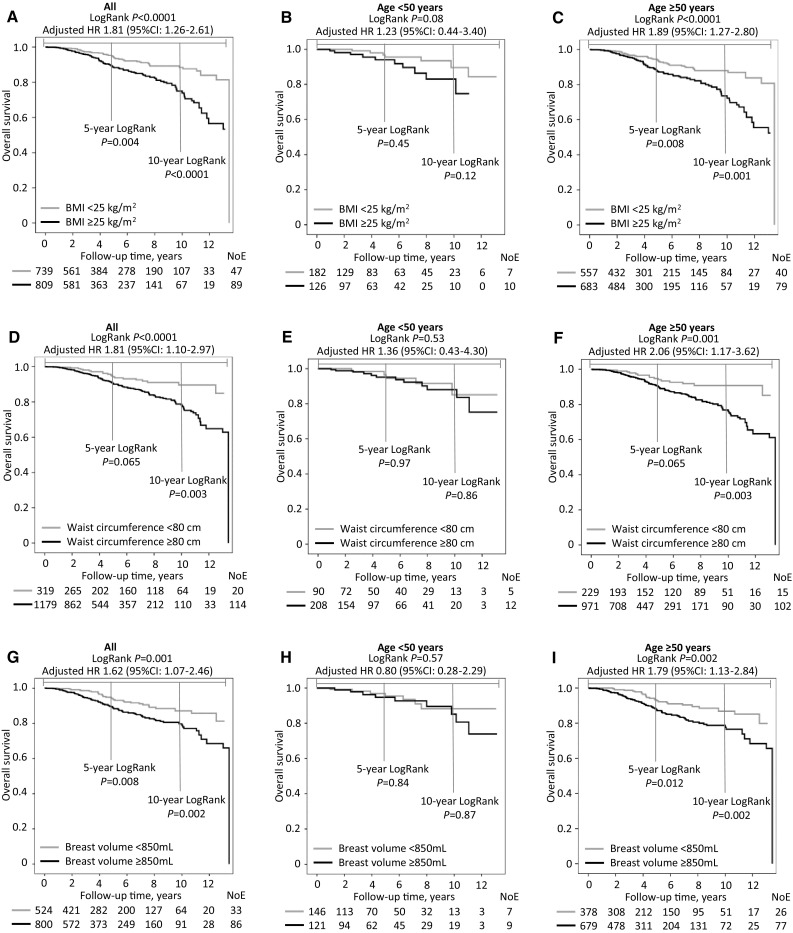
**a–i** BMI, waist circumference, and breast volume alone and stratified by age in relation to the overall survival. Because this is an ongoing cohort study, the number of patients decreased with each follow-up. HRs are presented with the 95% CIs and were adjusted for age at inclusion (continuous), invasive tumor size (< 21 mm vs. ≥21 mm, or skin or muscular involvement independent of size), any axillary lymph node involvement, histological grade III, and ER status (Model 1, Table 3)

The patients with a BMI ≥ 25 kg/m^2^, waist circumference ≥ 80 cm, and breast volume ≥ 850 mL had a shorter overall survival compared to patients with a smaller BMI, waist circumference, and breast volume (Fig. 3a, d, g). This association was significant in the older (Fig. 3c, f, i), but not the younger (Fig. 3b, e, h) patients. There were no significant effect modifications by age (all _adj_*p*_interactions_ ≥ 0.29), with the exception of the crude association between age and waist circumference ≥ 80 cm (*p*_interactions_ < 0.03; HR 1.93; 95% CI 1.06–3.52). When stratified by ER status, a BMI ≥ 25 kg/m^2^ was significantly associated with a shorter overall survival in patients with ER+ tumors (Log-Rank *p* < 0.0001; _adj_HR 1.91; 95% CI 1.26–2.91). This association was not seen in patients with ER− tumors (Log-Rank *p* = 0.17), although no significant effect modifications were found by ER status (_adj_*p*_interactions_≥0.28). A waist circumference ≥ 80 cm was associated with shorter overall survival in patients with ER+ (Log-Rank *p* = 0.007; _adj_HR 1.61; 95% CI 0.94–2.75), and ER− (Log-Rank *p* = 0.056; _adj_HR 3.54; 95% CI 0.83–15.22) tumors. A breast volume ≥ 850 mL was associated with overall survival in patients with ER + tumors (Log-Rank *p* = 0.001; _adj_HR 1.79; 95% CI 1.09–2.92), but not in patients with ER− tumors (Log-Rank *p* = 0.81).

**Table 3:** Overall survival by BMI, waist circumference, and breast volume and combinations of BMI, waist circumference, and breast volume

	Total	Events	Missing	Crude HR	Model 1	Model 2	Model 3		
	*n* 1,640	*n*	*n*	HR (95% CI)	_adj_HR (95% CI)^a^	_adj_HR (95% CI)^abc^	*n* 1,295	*n*	_adj_HR (95% CI)^abcd^
BMI < 25 kg/m^2^	739	47	92	Ref	Ref	Ref	621	37	Ref
BMI ≥ 25 kg/m^2^	809	89		**2.20 (1.54–3.15)**	**1.81 (1.26–2.61)**	**1.82 (1.24–2.65)**	674	74	**1.63 (1.00-2.64)**
Waist circumference < 80 cm	319	20	142	Ref	Ref	Ref	278	16	Ref
Waist circumference ≥ 80 cm	1179	114		**2.31 (1.43–3.72)**	**1.81 (1.10–2.97)**	**1.92 (1.16–3.18)**	1017	95	1.34 (0.70–2.54)
Breast volume < 850 mL	524	33	316	Ref	Ref	Ref	515	30	Ref
Breast volume ≥ 850 mL	800	86		**1.94 (1.30–2.90)**	**1.62 (1.07–2.46)**	**1.79 (1.16–2.76)**	780	81	1.31 (0.81–2.14)
Combinations of BMI and waist circumference			148						
BMI < 25 kg/m^2^ and waist circumference < 80 cm	305	18		Ref	Ref	Ref	266	14	Ref
BMI ≥ 25 kg/m^2^ and waist circumference < 80 cm	13	2		2.89 (0.67–12.48)	1.69 (0.39–7.39)	1.75 (0.39–7.88)	12	2	1.60 (0.34–7.50)
BMI < 25 kg/m^2^ and waist circumference ≥ 80 cm	403	28		1.66 (0.91–3.01)	1.35 (0.73–2.50)	1.51 (0.81–2.82)	355	23	1.33 (0.65–2.71)
BMI ≥ 25 kg/m^2^ and waist circumference ≥ 80 cm	771	85		**2.89 (1.74–4.82)**	**2.16 (1.27–3.67)**	**2.28 (1.32–3.92)**	662	72	**2.17 (1.11–4.23)**
Combinations of breast volume and BMI			322						
Breast volume < 850 mL and BMI < 25 kg/m^2^	410	19		Ref	Ref	Ref	406	17	Ref
Breast volume ≥ 850 mL and BMI < 25 kg/m^2^	217	20		1.86 (0.98–3.51)	1.59 (0.82–3.08)	1.82 (0.93–3.55)	215	20	1.67 (0.81–3.41)
Breast volume < 850 mL and BMI ≥ 25 kg/m^2^	111	14		**3.36 (1.68–6.72)**	**2.65 (1.29–5.43)**	**2.53 (1.19–5.39)**	109	13	2.22 (0.96–5.12)
Breast volume ≥ 850 mL and BMI ≥ 25 kg/m^2^	580	65		**3.17 (1.90–5.30)**	**2.59 (1.51–4.44)**	**2.76 (1.58–4.81)**	565	61	**2.40 (1.24–4.68)**
Combinations of breast volume and waist circumference			317						
Breast volume < 850 mL and waist circumference < 80 cm	229	11		Ref	Ref	Ref	226	10	Ref
Breast volume ≥ 850 mL and waist circumference < 80 cm	52	6		2.21 (0.82–5.97)	1.91 (0.69–5.27)	1.88 (0.67–5.26)	52	6	1.71 (0.60–4.82)
Breast volume < 850 mL and waist circumference ≥ 80 cm	295	22		**2.38 (1.15–4.91)**	1.97 (0.92–4.23)	1.94 (0.88–2.25)	289	20	1.54 (0.68–3.51)
Breast volume ≥ 850 mL and waist circumference ≥ 80 cm	747	79		**3.23 (1.72–6.07)**	**2.48 (1.27–4.83)**	**2.71 (1.37–5.34)**	728	75	1.90 (0.88–4.10)

Events (E) and missing data (MD), respectively, in the adjusted models; in Model 1: BMI 133 E, 10 MD, waist circumference 131 E, 10 MD, breast volume 116 E, 9 MD, in Model 2: BMI 130 E, 25 MD, waist circumference 128 E, 24 MD, breast volume 113 E, 22 MD

Bold numbers indicate significance with a *p*-value < 0.05

^a^Adjusted for age at inclusion (continuous), invasive tumor size (< 21 mm vs. ≥ 21 or skin or muscular involvement independent of size), any axillary lymph node involvement (yes), histological grade III (yes), and ER status

^b^Adjusted for alcohol abstention (yes) and current smoking prior to inclusion (yes)

^c^Adjusted for treatment; chemotherapy, radiotherapy, tamoxifen, AIs, trastuzumab

^d^Mutually adjusted for BMI ≥ 25 kg/m^2^, waist circumference ≥ 80 cm, and breast volume ≥ 850 mL

Among the single variables, patients with a BMI ≥ 25 kg/m^2^ or waist circumference ≥ 80 cm had a shorter overall survival than those with a breast volume ≥ 850 mL (Table 3). In the mutually adjusted Model 3, only the models including a BMI ≥ 25 kg/m^2^ remained significant.

### BMI, waist circumference, and breast volume in relation to prognosis in different treatment groups

Since anthropometric factors may impact certain treatment responses more than others, further analyses with stratification by treatments were conducted. A BMI ≥ 25 kg/m^2^ in patients ≥ 50 years with ER+ tumors was weakly associated with a shorter breast cancer-free interval in tamoxifen-treated (Log-Rank *p* = 0.032; _adj_HR 1.72; 95% CI 1.00–2.95) and AI-treated patients (Log-Rank *p* = 0.055; _adj_HR 1.71; 95% CI 0.91–3.20) compared to patients with a lower BMI. This difference was not seen in other treatment groups among all patients. Further, patients ≥ 50 years with ER+ tumors and a BMI ≥ 25 kg/m^2^ also had a shorter overall survival when treated with tamoxifen (Log-Rank *p* = 0.001; _adj_HR 2.28; 95% CI 1.29–4.03) or AIs (Log Rank *p* = 0.019; _adj_HR 1.75; 95% CI 0.92–3.30) compared to patients with lower BMI. For chemotherapy-treated patients, a high BMI was not significantly associated with overall survival. In contrast, in chemonaïve patients, a high BMI was associated with significantly shorter overall survival (Log-Rank *p* < 0.0001; _adj_HR 1.91; 95% CI 1.26–2.91) compared to patients with lower BMI.

For patients ≥ 50 years with ER+ tumors, a waist circumference ≥ 80 cm was associated with a shorter breast cancer-free interval if tamoxifen-treated (Log-Rank *p* = 0.048; _adj_HR 2.05; 95% CI 1.01–4.15) or AIs (Log-Rank *p* = 0.027; _adj_HR 2.82; 95% CI 0.99–8.04). In all patients, a waist circumference ≥ 80 cm was not associated with shorter disease-free survival if treated with chemotherapy, radiotherapy, or trastuzumab. In contrast, a waist circumference ≥ 80 cm was associated with shorter breast cancer-free interval if the patient was chemonaïve (Log-Rank *p* = 0.029; _adj_HR 1.55; 95% CI 0.98–2.45) or had not received radiotherapy (Log-Rank *p* = 0.003; _adj_HR 2.35; 95% CI 1.22–4.53). A waist circumference ≥ 80 cm was weakly associated with shorter overall survival in radiotherapy (Log-Rank *p* = 0.006; _adj_HR 1.79; 95% CI 0.93–3.45) and tamoxifen-treated patients (Log-Rank *p* = 0.030; _adj_HR 2.07; 95% CI 0.96–4.45) compared to lower waist circumference, but not in other treatment groups. A high waist circumference was significantly associated with shorter overall survival in chemonaïve patients (Log-Rank *p* = 0.001; _adj_HR 1.90; 95% CI 1.09–3.31).

A breast volume ≥ 850 mL was significantly associated with a shorter breast cancer-free interval for patients that received chemotherapy, radiotherapy, tamoxifen, AIs, and trastuzumab, in the univariable models (Supplementary Fig. 1A–E). These associations remained significant after adjustment for age at inclusion and tumor characteristics for chemotherapy and AI-treated patients. However, a breast volume ≥ 850 mL was also associated with shorter breast cancer-free interval in chemonaive patients (Log-Rank *p* = 0.056; _adj_HR 1.56; 95% CI 1.02–2.39) and in patients who had not received radiotherapy (Log-Rank *p* = 0.042; _adj_HR 1.90; 95% CI 1.10–3.29), compared to smaller breast volumes.

Breast volume ≥ 850 mL was associated with shorter overall survival in patients treated with radiotherapy (Log-Rank *p* = 0.004; _adj_HR 1.74; 95% CI 1.10–3.05) and tamoxifen (Log-Rank *p* = 0.009; _adj_HR 2.34; 95% CI 1.15–4.74) compared to smaller breast volumes, but not for other treatments. Large breast volume was significantly associated with shorter overall survival in chemonaïve patients (Log-Rank *p* = 0.005; _adj_HR 1.63; 95% CI 1.01–2.63).

### Restriction analyses

For Tables 2 and 3, restriction analyses were performed excluding the 345 patients for whom data were missing for one or more variables (except trastuzumab). The crude HRs and the _adj_HRs in Models 1 and 2 remained essentially the same for all anthropometric factors alone and combined, except for the combination of breast volume ≥ 850 mL and BMI < 25 kg/m^2^ shown in Table 3 that became significant with a crude HR of 2.10 (95% CI 1.09–4.03) for overall survival. Further, restriction analyses were performed with a cut-off age of 55 years with univariable and multivariable analyses of breast cancer-free interval and overall survival in relation to BMI, waist circumference, and breast volume and the results were materially the same as with cut-off age 50 years.

## Discussion

The main finding of this study in primary breast cancer patients was that preoperative body size measures, including BMI, waist circumference, and breast volume, significantly increased between 2002 and 2016. Breast volume was the strongest prognostic factor in terms of breast cancer-free interval, overall and in all treatment groups, while BMI and waist circumference were the strongest prognostic factors for overall survival.

The finding that breast volume and waist circumference are also important in addition to BMI is in line with previous studies and underlines the importance of including anthropometric factors that specifically measure body constitution when evaluating prognosis in the clinical setting [18]. One study found an association between increased WHR and increased breast cancer-specific mortality, as well as overall survival, and between increased waist circumference and higher all-cause mortality in breast cancer patients [19]. Previous studies have also shown that patients with larger breasts have shorter disease-free survival [20] and increased breast cancer mortality [21]. After statistical analyses of the impacts of WHR and waist circumference on prognostic markers in this study, the waist circumference was selected as a marker because it showed stronger effect estimates in relation to clinical outcome than WHR. One explanation could be that waist circumference may provide more information on total abdominal and visceral fat than WHR [28]. However, more research is needed to discern the mechanisms behind the association between increased anthropometric measurements and a poorer prognosis.

The differences found in the anthropometric factors in this study between patients with ER+ and ER− tumors and between younger and older patients contradict the findings of a meta-analysis, where pre-existing obesity was associated with poor prognosis regardless of menopausal or ER status [29]. Our results may be due to small number of patients < 50 years or with ER− tumors and there was no effect modification by tumor ER status and only in one of the crude analyses did we find an effect modification by age.

Several mechanisms could be responsible for our findings of a poorer prognosis for breast cancer patients with larger anthropometrics. It is known that obesity activates changes in the whole body as well as specifically in the breast that affect breast cancer progression. Obesity leads to higher levels of circulating insulin and IGF-1 in the body [14], which have been associated with risk for ER+ tumors [30] and increased mammographic density in women with BMIs < 25 kg/m^2^, but not in overweight and obese women [31]. Higher levels of circulating insulin increase the bioavailability of estrogen, a process associated with a poorer prognosis in breast cancer patients [32]. Increased ratios of adiponectin, leptin, and cytokines also promote inflammation, which creates a cancer-promoting environment [15, 33]; several cytokines are associated with poor breast cancer outcomes [34]. Obesity also leads to specific local changes in the breast. In obese patients, increased secretion of hormones such as leptin, as well as adipokines, contribute to the development of breast cancer. Increased aromatase production by adipocytes can also increase estrogen levels in the breast locally. Crown-like structures have been found around adipocytes that stimulate hypoxic conditions and enhance angiogenesis, which is a part of cancer development [14].

Regarding treatment, obesity may also undermine the response to endocrine therapy in premenopausal patients [35] and tamoxifen treatment in postmenopausal patients with ER+ tumors [33, 36]. The latter was confirmed in our study, where there was a significantly poorer prognosis for tamoxifen-treated patients ≥ 50 years old with ER+ tumors and BMIs ≥ 25 kg/m^2^, a waist circumference ≥ 80 cm, or a breast volume ≥ 850 mL. In line with that, a study conducted reported that overweight patients had a poorer prognosis compared to patients with normal weights, with anastrozole appearing to be more influenced by higher BMIs than tamoxifen [35]. However, a study analyzing the BIG-I-98 trial found no significant effect of BMI on treatment with letrozole or tamoxifen [37]. Chemotherapy, radiotherapy, and treatment with tamoxifen, AIs, and trastuzumab all lower the risk for recurrence and breast cancer mortality in breast cancer patients [38–41]. However, breast cancer patients may have an even better prognosis if they have a BMI < 25 kg/m^2^, a waist circumference < 80 cm, and a breast volume < 850 mL. A lower BMI may also equate to a smaller waist circumference and breast volume, but fat distribution varies between women. Weight management could contribute to a better treatment response for patients receiving tamoxifen and AIs [7].

According to the American Society of Clinical Oncology, correct chemotherapeutic dosing needs to be calculated by body weight instead of BMI to prevent under-dosing in obese patients [42]. National guidelines in Sweden recommend using body surface (m^2^) to calculate chemotherapy dosages [43]. However, higher chemotherapy dosing for overweight and obese patients can lead to severe side effects such as neurotoxicity [39], another reason that doses may need to be lowered or adjusted other than weight or body surface. Chemotherapy seemed to counteract the negative impact of a higher BMI or larger waist circumference in the patients in this study. In contrast, the patients with larger body sizes not treated with chemotherapy had a poor prognosis. It should be noted that patients not treated with one treatment such as chemotherapy may have been treated with radiotherapy, tamoxifen, AIs, or trastuzumab.

This study has some limitations. Treatment types and regimens have changed over time and may have affected the breast cancer-free interval and overall survival differently during the 2002–2016 time frame. The lower frequency of tamoxifen in the 2013–2016 patients might, in part, be due to the fact that they had not yet switched from AIs to tamoxifen treatment because the follow-up was less than 5 years for these patients. Another limitation of the study is that only preoperative data on alcohol consumption and current smoking were used. No information on co-morbidities or socioeconomic status was available. The power calculations for the study were sufficient for the main effects for the BMI, waist circumference, and breast volume, but the power to detect significant differences in subgroups was reduced.

A strength of the current study is that it is considered population-based, since the patients were not referred to other hospitals for surgery. In addition, it has been previously shown that the majority of patients who met the inclusion criteria of the BC-Blood Study were included. Non-participation was mainly due to a lack of available research nurses. With regard to age and ER status, the included patients were comparable to all women operated in Lund [44]. Body measurements were only self-reported for 2.5% of the patients. The vast majority of the exposure data were objectively measured by research nurses, which minimizes information bias.

Ninety-six patients with preoperative treatment were excluded from the present study. Compared to the enrolled patients, larger percentages were younger than 50 years and had ER− tumors, so their inclusion could have affected the results. Because the body measurements were obtained after preoperative treatment, they could have changed for the excluded patients, as weight gain and changes in body composition have previously been reported in breast cancer patients during adjuvant therapy [45]. Thus, their measurements would not be representative of the patients included in this study. Nevertheless, the association between age at inclusion and tumor ER status remained insignificant even when the 96 patients were included (data not shown).

The data as a whole indicated increased body size is associated with a worse clinical outcome. Therefore, lifestyle programs that target weight reduction could be incorporated into the care of breast cancer patients, something not currently included in clinical practice [15].

In conclusion, in this study, preoperative body measurements were assessed in primary breast cancer patients between 2002 and 2016 and investigated in relation to clinical outcomes. Over time, the proportion of patients with higher BMIs, waist circumferences, and breast volumes significantly increased. In terms of clinical outcome, breast volume was the strongest prognostic and predictive factor associated with the breast cancer-free interval. BMI and waist circumference were the strongest prognostic factors with respect to overall survival. If confirmed, measurements of body constitution could help guide the selection of treatment for each patient.

## Electronic supplementary material

Below is the link to the electronic supplementary material.

Supplementary material 1. Supplementary Figure 1. (**A–E**) Kaplan-Meier estimates of the breast cancer-free interval in relation to breast volume in all patients treated with adjuvant chemotherapy (**A**) or radiotherapy (**B**), in patients ≥50 years of age and with ER+ tumours treated with tamoxifen (**C**), or AIs (**D**), and in patients treated with trastuzumab as of November 2005 (**E**). Because this is an ongoing cohort study, the number of patients decreased with each follow-up. HRs are presented with the 95% CIs and were adjusted for age at inclusion (continuous), invasive tumour size (<21mm vs. ≥21 or skin or muscular involvement independent of size), any axillary lymph node involvement, histological grade III, and ER-status. (PDF 307 KB)
