# Supplementary figures and images for: Increasing preoperative body size in breast cancer patients between 2002 and 2016: implications for prognosis
Source: Cancer Causes Control. 2018 May 26;29(7):643–56. doi: 10.1007/s10552-018-1042-z (PMC5999186; doi:10.1007/s10552-018-1042-z)

Supplementary figure 1

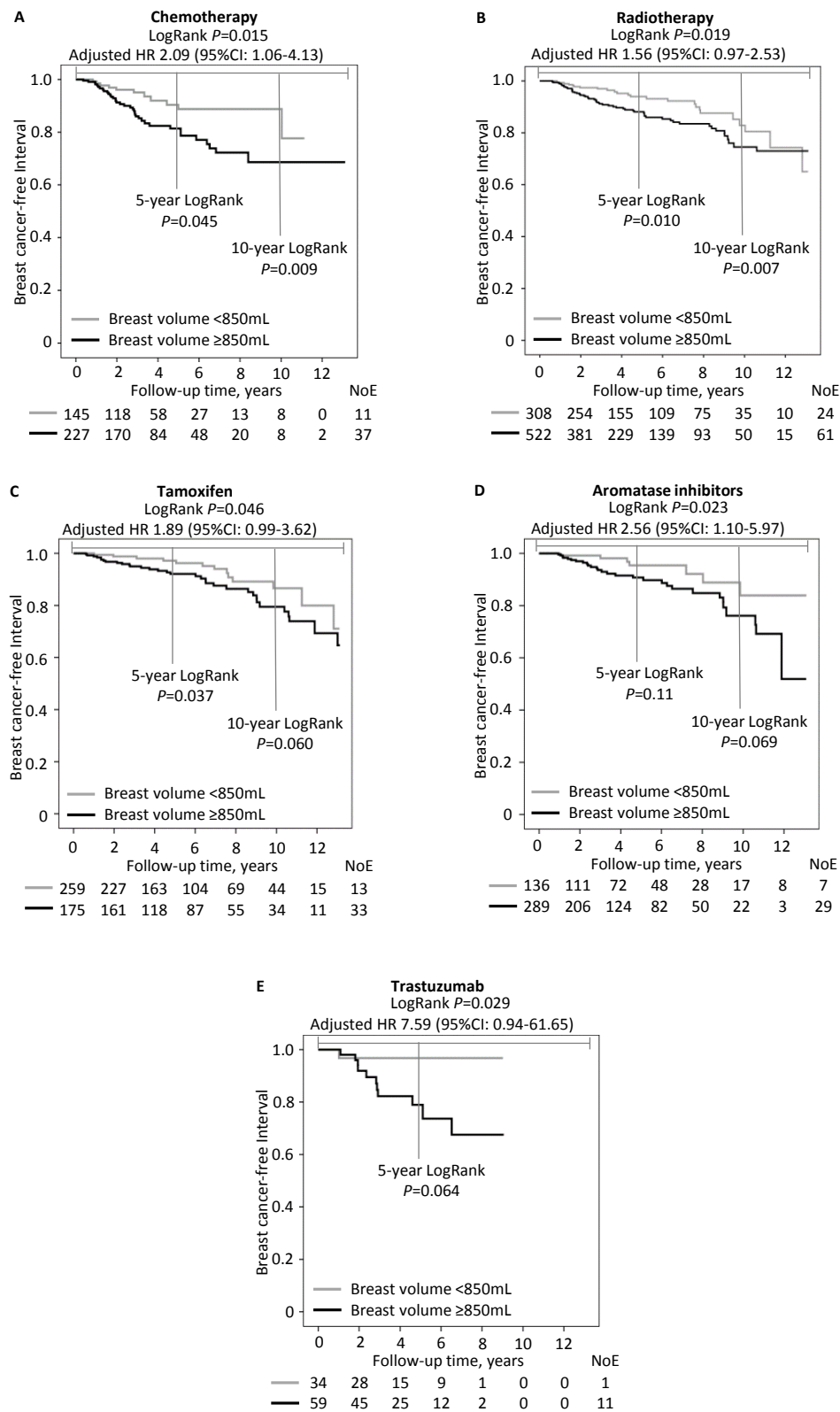

Supplement: Supplementary file 1 — Supplementary material 1. Supplementary Figure 1. (A–E) Kaplan-Meier estimates of the breast cancer-free interval in relation to breast volume in all patients treated with adjuvant chemotherapy (A) or radiotherapy (B), in patients ≥50 years of age and with ER+ tumours treated with tamoxifen (C), or AIs (D), and in patients treated with trastuzumab as of November 2005 (E). Because this is an ongoing cohort study, the number of patients decreased with each follow-up. HRs are presented with the 95% CIs and were adjusted for age at inclusion (continuous), invasive tumour size (<21mm vs. ≥21 or skin or muscular involvement independent of size), any axillary lymph node involvement, histological grade III, and ER-status. (PDF 307 KB) [file 10552_2018_1042_MOESM1_ESM.pdf]
